# L-Glutamine attenuates peritoneal fibrosis developed in 5-Fluorouracil-treated mice

**DOI:** 10.3389/ebm.2026.10755

**Published:** 2026-02-24

**Authors:** Juliana Francisca Grossi Heleno, Leticia Cristine Cardoso dos Santos, Igor Campos Fontes, Mirielly Ranny Almeida Paiva Silva, Lucas Barbosa Correia, Nayma Drielly Granato Silva, Pedro Henrique Dias Moura Prazeres, Pedro Pires Goulart Guimarães, Derek W. Gilroy, Silvia Passos Andrade, Paula Peixoto Campos

**Affiliations:** 1 General Pathology Department, Biological Science Institute, Federal University of Minas Gerais, Belo Horizonte, Brazil; 2 Benjamin Guimarães Foundation, Hospital of Baleia, Department of Hospital Dentistry, Belo Horizonte, Minas Gerais, Brazil; 3 Department of Physiology and Biophysics, Institute of Biological Sciences, Federal University of Minas Gerais, Belo Horizonte, Minas Gerais, Brazil; 4 Department of Ageing, Rheumatology and Regenerative Medicine, University College London, London, United Kingdom

**Keywords:** angiogenesis, fibrogenesis, immunonutrient, inflammation, repair

## Abstract

Peritoneal fibrosis is an adverse effect of cancer therapy leading to progressive organ failure. L-Glutamine supplementation has been shown to attenuate fibrosis and improve wound healing in several types of tissue injuries. The aim of this study was to evaluate the effects of this supplementation on key components of the peritoneal fibrovascular tissue induced by implants in mice treated with 5-Fluorouracil (5-FU) C57BL/6 mice received three intraperitoneal doses of immunosuppressant (60, 40, and 40 mg/kg) on non-consecutive days prior to implantation of polyether-polyurethane sponges into the peritoneal cavity. The group treated with L-Glutamine received 150 mg/kg/day for 7 days (oral gavage) starting 24 h after implantation and the control group received filtered water. Eight days after implantation, implants were removed and processed for inflammatory, angiogenic, and fibrogenic markers. Flow cytometry results showed that L-Glutamine decreased (48%) the frequency/influx of total intra-implant cells. The remaining cell population in the treated group had more neutrophils, lymphocytes, and macrophages than in the control. Immunohistochemistry analysis showed fewer Caspase-3-positive cells in the treated group. Myeloperoxidase (MPO) and N-acetyl-β-D-glucosaminidase (NAG) activities, TNF-α levels, and mast cell numbers were decreased in the implants of the L-Glutamine-treated group compared with the control. Similarly, angiogenesis (VEGF levels and number of blood vessels) was attenuated by L-Glutamine. Supplementation also decreased the amount of intra-implant collagen and TGF-β1 levels. These results indicate that L-Glutamine attenuates critical inflammatory-angiogenesis and profibrotic pathways involved in fibrosis development in immunosuppression conditions, supporting its potential as an adjunct therapeutic strategy for managing peritoneal healing in cancer.

## Impact statement

Adverse healing (fibrosis/adhesion) in the peritoneal cavity occurs after traumatic surgical procedures, injection and cancer treatments, impairing the function of visceral organs. Effective prevention and therapeutic management strategies are needed, especially under immunosupression. L-Glutamine (GLN), an aminoacid used as a nutritional supplement, has been shown to modulate inflammation, angiogenesis and fibrosis, key components of the wound healing process. Our data provides important information on the effects of oral L-Glutamine supplementation on the development of fibrovascular tissue in a model of implant induced peritoneal adhesion in mice treated with 5-Fluoracil (5-FU). The aminoacid was able to differentially modulate the recruitment/activation of inflammatory cells of the myeloid lineage towards a predominantly anti-inflammatory, anti-angiogenic and anti-fibrogenic phenotype on the peritoneal fibrovascular tissue under immunosupression. By targeting multiple axes involved in peritoneal fibrosis, our findings advance the knowledge on the mechanisms of action of L-Glutamine controlling wound healing in the abdominal cavity.

## Introduction

Peritoneal adhesions are characterized by the abnormal formation of fibrous connections between tissues and organs within the abdominal cavity due to dysregulated wound healing processes such as excessive collagen deposition and inflammatory cytokine release. They develop in 75%–90% of patients following abdominal surgery, infection, irradiation, or the introduction of foreign materials like surgical sutures and meshes [1–3]. These adhesions can lead to significant health complications, including bowel obstruction, chronic abdominal pain, and female infertility, resulting in substantial economic burdens due to increased hospitalizations, repeat surgical interventions, and reduced patient productivity [3–6]. Peritoneal injury disrupts the mesothelial layer triggering a cascade of events such as coagulation, inflammation, fibrinolysis, angiogenesis, extracellular matrix deposition. These events are orchestrated by an influx of various cell types, immune cells, endothelial cells, mast cells and stromal fibroblasts that, in turn release their products at the site of the injury resulting in a fibrous exudate [4–7]. When fibrinolytic imbalance occurs, this exudate may develop into fibrous tissue, as mesothelial cells, fibroblasts, and myofibroblasts contribute to the extracellular matrix deposition, accompanied by the prolonged activation of inflammatory and angiogenic processes [4–7]. The newly formed tissue is a dysmorphic connective structure composed of a dense, disorganized extracellular matrix, highly vascularized, differentiated, innervated, and populated with a variety of cell types [4–7]. This adverse healing process (fibrosis/abdominal adhesion) is responsible for impairing the functionality of visceral organs, constituting a major cause of morbidity and mortality [1–3]. Despite ongoing research, the precise mechanisms underlying adhesion formation remain incompletely understood, and effective preventive and therapeutic strategies are still needed [8, 9].

L-Glutamine, the most abundant free amino acid in the human body, is a crucial cellular substrate, not only as an amino acid, but also as a source of energy, nitrogen and carbon for macrophages, lymphocytes and other cells (enterocytes, fibroblasts) [10–13]. Thus, L-Glutamine is directly involved in the process of immunological cell division, in acid-base balance, in the transport of ammonia between tissues, in the donation of carbon skeletons for gluconeogenesis and participates in the processes of recovery from physiological stress due to harmful and/or catabolic events [10, 14]. The reduction in the availability of L-Glutamine appears to influence several functions, whether in the immune system, antioxidant action or inflammation induced by physical exercise or disease. Other studies have attributed the improvement of tissue repair processes in wounds with increased catabolism to oral supplementation with L-Glutamine, such as in seriously ill, burned, cancer or critical patients, where L-Glutamine consumption exceeds the body’s synthesis capacity [13, 15]. In 1999, it was reported that total parenteral nutrition with glutamine shortened hospital stays and improved immunity after major abdominal surgery [16]. However, the effects of this supplementation are far from clear cut. It has been reported that this amino acid exerted both anti- and pro- inflammatory, angiogenic and fibrogenic effects on various damaged/injured tissues, with improvement in the quality of remodeling [17–21]. For instance, it has been reported that inhibition of L-Glutamine metabolism by an analogue, 6-diazo-5-oxo-L-norleucine, accelerated the resolution and repair of acute lung injury induced by intratracheal lipopolysaccharide (LPS) instillation [18]. In contrast, L-Glutamine supplementation was able to decrease the expression of various pro-inflammatory mediators in rat osteoarthritis [17, 18]. In the angiogenic process, glutamine has been shown to play a key role in endothelial cell sprouting and blood vessel formation [19, 20, 22].

However, different results have been obtained for migration experiments performed under glutamine withdrawal, depending on the endothelial cell types used [19, 20, 22]. Glutamine has been shown to either down or up regulate fibrosis in several experimental/human conditions. The use of L-Glutamine enema reduced inflammation and fibrosis in experimental diversion colitis in Wistar rats [23]. Oral administration of glutamine promoted faster skin healing by acting on several stages of healing, such as collagen synthesis, wound contraction and epithelialization in a rat wound model [24]. However, pro-fibrotic effects of L-Glutamine have been reported. Patients with severe fibrosis exhibit elevated serum glutamine levels and increased expression of kidney glutamine synthetase. Deprivation of glutamine metabolism *in vitro* and *in vivo* inhibits fibroblast activation, ameliorating renal fibrosis [22]. Furthermore, inhibition of glutamine metabolism suppressed activation of hepatic stellate cells attenuating liver fibrosis [25]. Thus, further investigation is needed to advance our knowledge on the effects of glutamine on various components of fibroproliferative conditions (inflammation, angiogenesis and fibrogenesis).

We hypothesized that this amino acid might modulate these components on our murine model of peritoneal fibrosis induced by the synthetic matrix of polyether-polyurethane. In this model, surgical implantation of the matrix elicited the formation of an adhesion-like tissue in which inflammation, angiogenesis and fibrosis are identified [26–29]. Given that L-Glutamine supplementation is recommended to patients undergoing chemotherapy, and this population is prone to have simultaneous medical interventions and/or tissue damage (surgery, tissue replacement, fibrosis), the animals in this study were also treated with 5-FU. This drug is one of the most widely employed antimetabolite chemotherapeutic used as a first line antineoplastic agent in the treatment of several cancers in humans, such as colorectal, breast, head and neck, pancreas and stomach cancers [30]. The reported adverse side effects of this drug in humans and experimental animal models, includes immunosuppressive and catabolic actions that directly influence wound repair processes [31, 32]. Due to these actions, this drug has been extensively used as a pharmacological model/tool to study several types of injuries and diseases in experimental animals and *in vitro* systems [31–33]. This study is the first, to our knowledge, to investigate the effects of L-Glutamine on peritoneal fibrosis in immunosuppressed animals.

## Materials and methods

### Animals

In this study, male C57BL/6 mice (7–8 weeks old weighing 17–21 g, n = 25 per group) were obtained from the Animal House (Bioterio Central) of Federal University of Minas Gerais (UFMG)-Brazil. The animals in each experimental group were allocated to distinct and non-overlapping analyses, including flow cytometry (10 animals), biochemical assays and cytokines (10 animals), and histological evaluation (5 animals), according to the specific processing requirements of each tissue. The mice were housed individually, with free access to standard chow and water, and maintained at 12:12 h light/dark cycle (lights on at 7:00 a.m.). To ensure ethical treatment, all procedures were designed to minimize animal distress and were performed in compliance with the UFMG Institutional Animal Committee guidelines (CEUA no 204/2022).

### Administration of chemotherapeutic drug (5-FU)

The animals were prepared to receive intraperitoneal injections of 5-FU for 3 non-consecutive days (at the doses of 60 mg/kg, on day 1 and 40 mg/kg on days 3 and 5). These days, in the timeline of the experiment, they were denoted as day −5, −3 and −1, respectively. The doses were chosen according to the protocol established by Sonis et al., 1990 and 2004 [31, 33]. Before 5-FU administration the animals were weighed and blood samples collected for quantitative determination of leukocytes, which was used as a reference to establish leukopenia at the time of euthanasia (n = 10 for each group). An overdose of the anesthetics (10 x the anesthetic dose) was used to euthanize the animals. At the time of euthanasia (day 13 after the first dose of 5-FU) an overdose of the anesthetics (10 x the anesthetic dose), blood samples were collected to confirm leukopenia (n = 10) and body weight evaluated.

### Implantation of synthetic matrix implants

Polyether-polyurethane sponges (Vitafoam Ltd., Manchester, UK) were used as the implanted material. The implants were disc-shaped (cut from a plate using a surgical punch) measuring 5 mm in thickness and 8 mm in diameter. Prior to surgery, the sponge discs were immersed in 70% v/v ethanol overnight and subsequently sterilized by boiling in distilled water for 30 min. Anesthesia was induced using a bolus of ketamine (150 mg/kg) and xylazine (10 mg/kg). The ventral hair was shaved and the exposed skin wiped with 70% v/v ethanol. The sponge discs were aseptically implanted intraperitoneally, through a 1-cm long ventral midline incision. Post-operatively, the animals were monitored for any signs of infection at the surgical site, discomfort, or distress; any animal showing such signs was promptly euthanized.

### Administration of oral L-Glutamine

The treated group received L-Glutamine at a dose of 150 mg/kg/day, diluted in 100 µL of filtered water (oral gavage) once daily for 7 consecutive days, starting the day after surgery. The dosage was selected based on previously published experimental studies in animals [31, 33]. The control group received an equal volume of filtered water (oral gavage) for the same duration. Animals were withheld from food and water for 30 min to prevent interference with substance absorption.

### Histological analysis

Implants from each group of mice (n = 5) were carefully excised, dissected free of adherent tissue, and fixed in formalin (10% v/v in isotonic saline) and used for all histological analyses. Sections (5 µm) were stained with Hematoxylin and Eosin (H&E) for evaluation of the fibrovascular tissue, inflammatory cells counting and blood vessels determination. Images of 20 fields per slide of each implant were captured for data analysis with an objective lens (×40) on an optical microscope (final magnification of ×400). Dominici staining (a mast cell marker) was employed to quantify this cell population. A countable mast cell was defined as a structure with metachromasia. Images of 20 fields from histological cross‐sections from each implant were captured with a panchromatic objective lens (×40) in an optical microscope (final magnification ×400).

Picrosirius-red staining was used followed by polarized light microscopy to identify and quantify collagen fibers. To carry out this analysis, images of sequential cross-sections of each implant were obtained from 20 fields, and images of histological cross-sections (area = 4,795 μm^2^) of the implants were captured using a panchromatic objective lens (×40) on an Olympus BX43 light microscope (final magnification = ×400).

All the images were digitized and analyzed using Image Pro-Plus 7.0 software with a final resolution of 2,560 × 1920 pixels (Media Cybernetics Inc., USA).

### Immunohistochemistry (IHC)

IHC reaction for the detection of Caspase-3 was performed using mouse anti-primary antibody Caspase-3 (1:300; #9662 Cell Signaling). Tissue sections (5 μm) were dewaxed, and a heat-induced antigen retrieval was performed using a pressure cooker with citrate buffer (pH 6), the slides were then cooled for 20 min in the same buffer. Sections were incubated for 15 min with Peroxidase Blocker from Novolink Polymer Detection Systems (Leica) and protein blocking was performed with solution from the same IHC kit. The slides were then incubated overnight at 4 °C with the primary antibody: caspase (1:300); followed by incubation with Post Primary and Polymer solutions. Peroxidase activity was visualized using DAB chromogen. The absence of staining in negative controls, where the primary antibody was omitted, confirmed specificity. Sections were then counterstained with hematoxylin. Protein expression was quantified by measuring the stained area within the implants, regardless of staining intensity. For morphometric analysis, images of stained cells from all cross-sectional fields using a ×100 planapochromatic objective (final magnification = ×1000) in light microscopy. The Caspase-3 images were digitized through an Olympus microcamera and transferred to an analyzer (QCapture). The results are presented as the number of Caspase-3 positive cells per field.

### Flow cytometry analysis

Intra-implant leukocytes were quantified by flow cytometry. The following monoclonal panel of fluorescent antibodies were used: anti-CD45 (fluorochrome Pe-Cy5), anti-CD11c (fluorochromeViviD/eF450), anti-CD3 (fluorochrome PE), anti-F4/80 (fluorochrome FITC), anti-MHC-ll (fluorochrome Cy7PE), anti-GR-1 (fluorochrome APC). Ten implants from each group were evaluated. The implants were shred with scissors in 1 mL of HBSS, then 2.5 mL of filtered and sterilized type 1 collagenase (Sigma Chemicals, St Louis, MO, USA) and trypsin were added to the fragments. After incubation for 30 min at 37 °C, the cells were washed and centrifuged (500 g for 10 min at 4 °C). The cells isolated from implants were suspended in a staining buffer (0.1% BSA in PBS). Subsequently, the cells were washed with FACS buffer, flushed to surface molecules for 20 min at 4 °C. At least 50,000 blocked - events were acquired for analysis using FACSCanto-ll (BD Biosciences, San Jose, CA, USA).

The data were analyzed using FlowJo Version 9.7.5 (TreeStar, Carrum Downs, Australia). Direct dispersion (FSC-A) and lateral dispersion (SSC-A) were used to initially remove debris and capture leukocytes. Leukocytes were quantified based on CD45 expression, and then T lymphocytes were quantified based on CD45 + CD3 expression. From the CD45^+^ cells, the expression of GR1 versus F4/80 was evaluated to select monocytes (GR1 + and GR1Low) and neutrophils (GR1 + F4/80Neg). The F4/80 High GR1Low/Neg port was used to characterize macrophages.

### Determination of myeloperoxidase and N-acetyl-β-DGlucosaminidase activities

Neutrophil infiltration into the implants was assessed indirectly by analyzing MPO activity, a lysosomal hemoprotein found in the azurophilic granules in neutrophil as previously described [34, 35]. Pellets from centrifugation of implants homogenates were divided into two portions, a part of the corresponding pellet was weighed, homogenized in pH 4.7 buffer (0.1M NaCl, 0.02M NaPO_4_, 0.015M NaEDTA), and centrifuged at 12,000 × g for 10 min. The pellets were then resuspended in 0.05M NaPO_4_ buffer (pH 5.4) containing 0.5% hexadecyltrimethylammonium bromide (HTAB) followed by three freeze thaw cycles using liquid nitrogen. MPO activity in the supernatant samples was assayed by measuring the change in absorbance (optical density; OD) at 450 nm using tetramethylbenzidine (1.6 mM) and H_2_O_2_ (0.3 mM). The reaction was terminated by adding 50 mL of H_2_SO_4_ (4M). Results were expressed as a change in OD per g of wet tissue. No standard curve was used; therefore, this represents a semi-quantitative measure of MPO activity. The remaining part of the pellet was used to quantify the extent of mononuclear cells accumulation in the implants by measuring the levels of the lysosomal enzyme NAG present in high levels in activated macrophages [34–36]. Briefly, these pellets were weighed, homogenized in NaCl solution (0.9% w/v) containing 0.1% v/v Triton X-100 (Promega, Madison, WI, USA), and centrifuged (3000 × g; 10 min at 4 °C). Samples (100 μL) of the resulting supernatant were incubated for 10 min with 100 μL of p-nitrophenyl-Nacetyl-β-D-glucosaminide (Sigma-Aldrich, St. Louis, MO, USA) prepared in citrate/phosphate buffer (0.1M citric acid, 0.1M Na_2_HPO_4_; pH 4.5) to yield a final concentration of 2.24 mmol. The reaction was stopped by the addition of 100 μL of 0.2-M glycine buffer (pH 10.6). Hydrolysis of the substrate was determined by measuring the absorption at 400 nm. Results are expressed as nmol per g of wet tissue.

### Measurement of TNF-α, VEGF and TGF-β1 production

In the remaining supernatants of the implants used for inflammatory enzymes, production of the cytokines were evaluated (n = 10). They were homogenized in PBS pH 7.4 containing 0.05% Tween, and centrifuged at 10,000 x g for 30 min. The levels of the cytokines in the supernatant from each implant (50 µL) were measured using Immunoassay Kits (R and D Systems, USA) and following the manufacturer’s protocol to each cytokine. Briefly, dilutions of cell-free supernatants were added in duplicate to ELISA plates coated with a specific murine monoclonal antibody against cytokine, followed by the addition of a second horseradish peroxidase-conjugated polyclonal antibody, also against cytokine.

After washing to remove any unbound antibody-enzyme reagent, a substrate solution (50 µL of a 1:1 solution of hydrogen peroxide and tetramethylbenzidine 10 mg/mL in DMSO) was added to the wells. Color development was halted after 20 min incubation with 2M sulfuric acid (50 µL), and the intensity of the color was measured at 540 nm on a spectrophotometer (Thermoplate). Standards were 0.5-log10 dilutions of recombinant murine cytokines from 7.5 ρg mL^−1^ to 1000 ρg mL^−1^ (100 µL). The threshold of sensitivity for each chemokine is 15.625 ρg/mL. The results were expressed as ρg cytokine per mg wet tissue.

### Statistical analysis

All data are presented as mean ± SEM for normally distributed variables. The normality of the data was assessed using the Shapiro–Wilk test, and homogeneity of variances was evaluated with Levene’s test. When the assumptions of normality and homoscedasticity were met, parametric tests were applied (Student’s *t*-test for comparisons between two groups, two-way ANOVA followed by appropriate post-hoc tests for multiple groups). Data are presented as mean ± SEM, and p < 0.05 was considered statistically significant. Outliers were identified and excluded using the ROUT method (Q = 1%) through the “Identify Outliers” function in GraphPad Prism 8.2.1 (GraphPad Software, San Diego, CA, USA).

## Results

**FIGURE 1:**
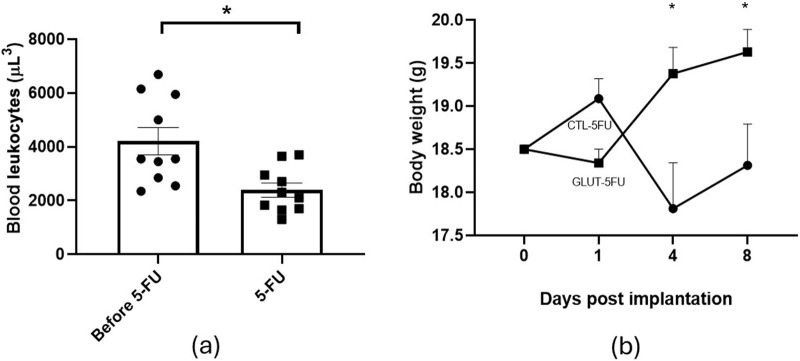
Effects of 5-FU on blood leukocytes number and body weight. Administration of the chemotherapeutic agent caused a decrease in blood leukocytes (48.8%) compared with the samples before 5-FU administration **(a)**. There was a progressive weight gain in the L-Glutamine treated group compared with a loss in body weight was observed in the control group **(b)**. Values are means ± SEM of 10 animals in each group. *p < 0.05. Body weight over time and between groups was analyzed by two-way ANOVA, while paired leukocyte counts before and after treatment were analyzed by paired Student’s t-test.

The protocol used to induce immunosuppression (3 doses of 5-FU) resulted in a decrease in the number of leukocytes, p = 0.014 (n = 10). The initial counting in the blood samples, before 5-FU, was 4,209 ± 506 leukocytes. On day 8 post implantation, when the animals were euthanized, the leukocyte number was 2,388 ± 265 (Figure 1a). In relation to body weight (Figure 1b), there was a significant difference between the animal’s weight of the two groups. The control group lost more weight compared with the treated group (p = 0.014 days 4 and p = 0.098 days 8) (n = 10). Oral administration of L-Glutamine (150 mg/kg/day) did not show signs of toxicity, dietary restrictions or changes in the animals' motor activity. The synthetic matrix was well tolerated by all animals. No signs of infection or rejection were observed at the implant site during the period of the experiment.

### Histopathological analysis - macro and microscopic evaluation of the implant

**FIGURE 2:**
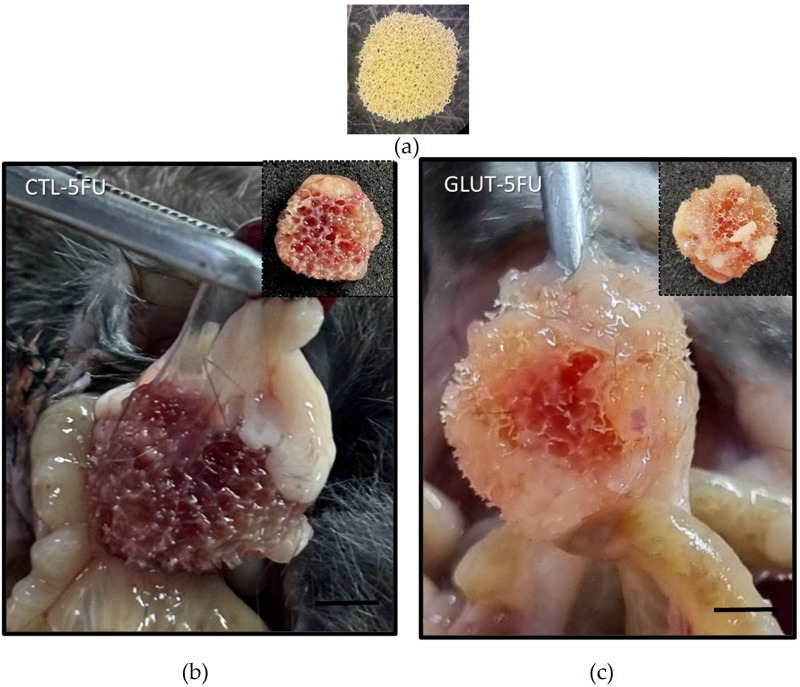
*In situ* images of polyether–polyurethane intraperitoneal implants. Image of the sponge disc before implantation **(a)**. Representative images of implants adhered to intestines through a band of fibrous tissue and removed from the peritoneal cavity at day 8 (Control **(b)**; Treated **(c)**). Scale bar, 50 μm.

**FIGURE 3:**
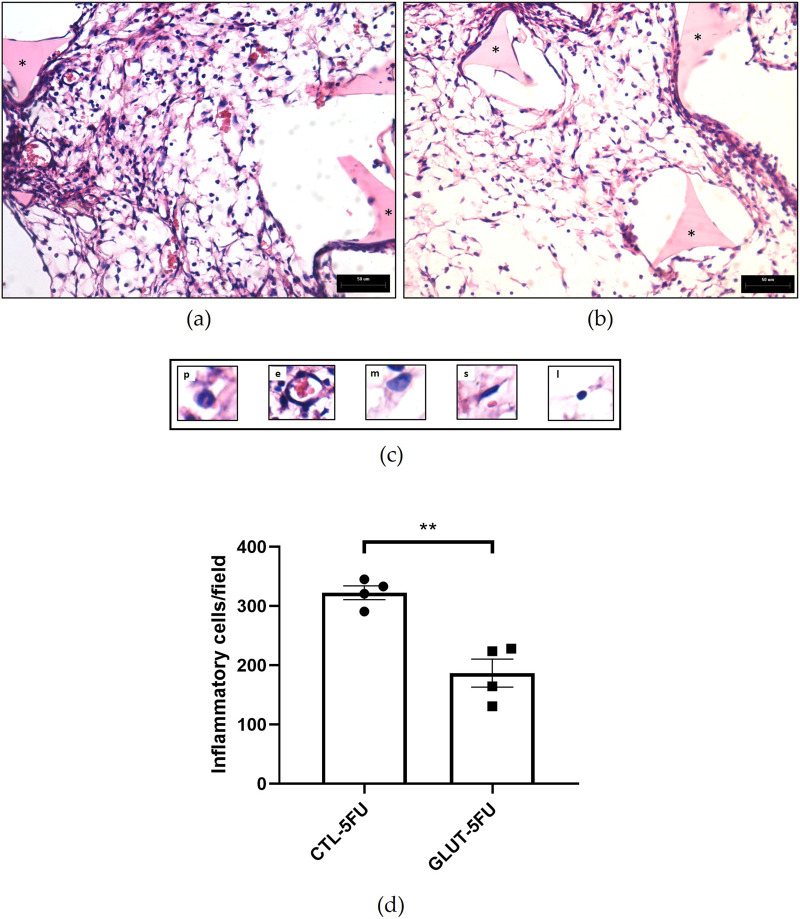
Representative histological sections of intraperitoneal implants stained with Haematoxylin and Eosin (H&E). In the fibrovascular tissue a greater number of cells and vessels are seen in implants of control mice compared with the implants of treated animals (control **(a)**; treated **(b)**). Different cell types were identified morphologically: polymorphonuclear cells (p), endothelial cells (e), mononuclear cells (m), spindle cells (s) and lymphocytes (l). The number of cells in control is higher than in the treated group **(c)**. Inflammatory cells **(d)** counting (n=5) were lower in the implants after the treatment. *Sponge matrix, arrows; blood vessels. Values are means ± SEM of five animals in each group. **p < 0.001, Student unpaired t-test. Planapochromatic objective (×40) in light microscopy (final magnification = ×400). Scale bar, 50 μm.

*In situ* images of the implants showed that they were firmly fixed in abdominal organs, liver and intestines. In the macroscopic analysis, the implants in the control group apparently showed a greater quantity of red blood cells, visibly evidenced by the color of the implants (Figures 2a–c). Histological analysis (H&E staining) was employed to evaluate the fibroproliferative tissue formed within and around the synthetic matrix. In both groups (control and treated), it was observed that the newly formed tissue was composed of inflammatory cells, blood vessels, extracellular matrix and an eosinophilic transudate (Figures 3a,b). However, in the control group the number of these features was higher. The mean number of inflammatory cells per field (n = 5) in the control group was 322 ± 11 versus 186 ± 23, p = 0.029 in the treated group (Figure 3c).

### Flow cytometry analysis

**FIGURE 4:**
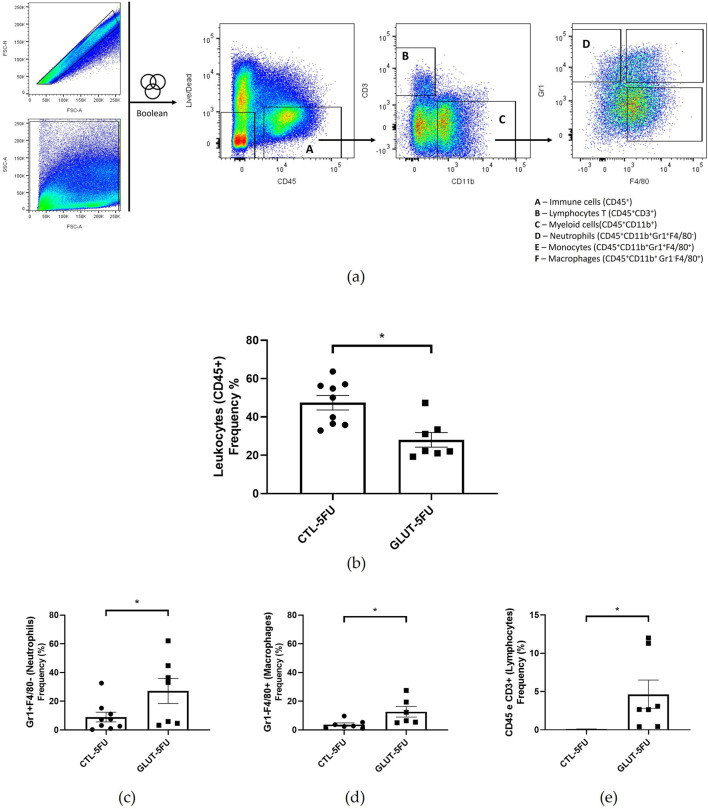
Effects of L-Glutamine on the recruitment of different cell types intra-implant (flow cytometry). Represented in **(a)** is the strategy of analysis in dot plots identifying the infiltration of neutrophils, macrophages and T lymphocytes, inside the implant. In **(b)**, the frequency of total immune cells and **(c–e)** the total count of neutrophils, macrophages and lymphocytes were compared in both groups, and the results are expressed as the mean ± SEM. *p < 0.05; Student’s unpaired t-test, n = 7-9 animals per group.

Using flow cytometry, we were able to analyze specific populations of inflammatory cells in the fibrovascular tissue. Our results showed that L-Glutamine was effective in reducing the overall frequency of CD45^+^ cells (40.87%; p = 0.031). However, when immune cell subsets were analyzed as relative frequencies with the CD45^+^ population, L-Glutamine group exhibited higher proportions of neutrophils (3-fold; p = 0.046; n = 7–9), macrophages by (3-fold; p = 0.032; n 7-9, and lymphocytes by (5-fold; p = 0.014; n = 7–9), than control group (Figures 4a–e).

### Immunohistochemistry for Caspase-3

**FIGURE 5:**
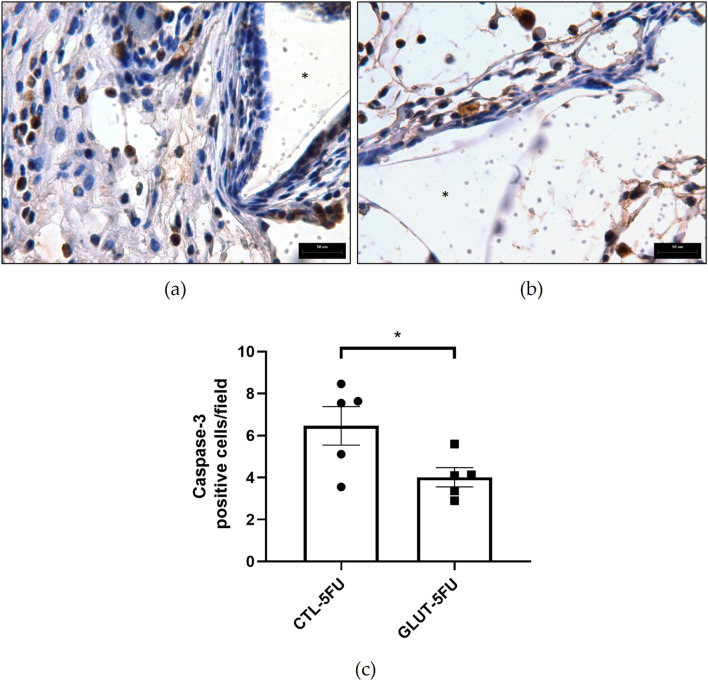
Effect of L-Glutamine on the apoptotic marker. Representative micrographs of intraperitoneal implants (**(a)**, control; **(b)**, treated) immunostained with Caspase-3. More positive cells were observed in the control implant compared with the treated **(c)**. Values are means ± SEM; *p < 0.05, Student’s unpaired t-test, n = 5. Planapochromatic objective (×100) in light microscopy (final magnification = ×1000). Scale bar, 50 μm.

The number of Caspase-3 positive cells (marked in brown) was higher in the control group 6.5 ± 0.9 compared with the treated group 4.0 ± 0.5, p = 0.044 (n = 5 for both groups). This indicates that L-Glutamine supplementation exerted an anti-apoptotic effect in our adhesion model (Figures 5a–c).

### Measurement of MPO, NAG, TNF-alpha and mast cells

**FIGURE 6:**
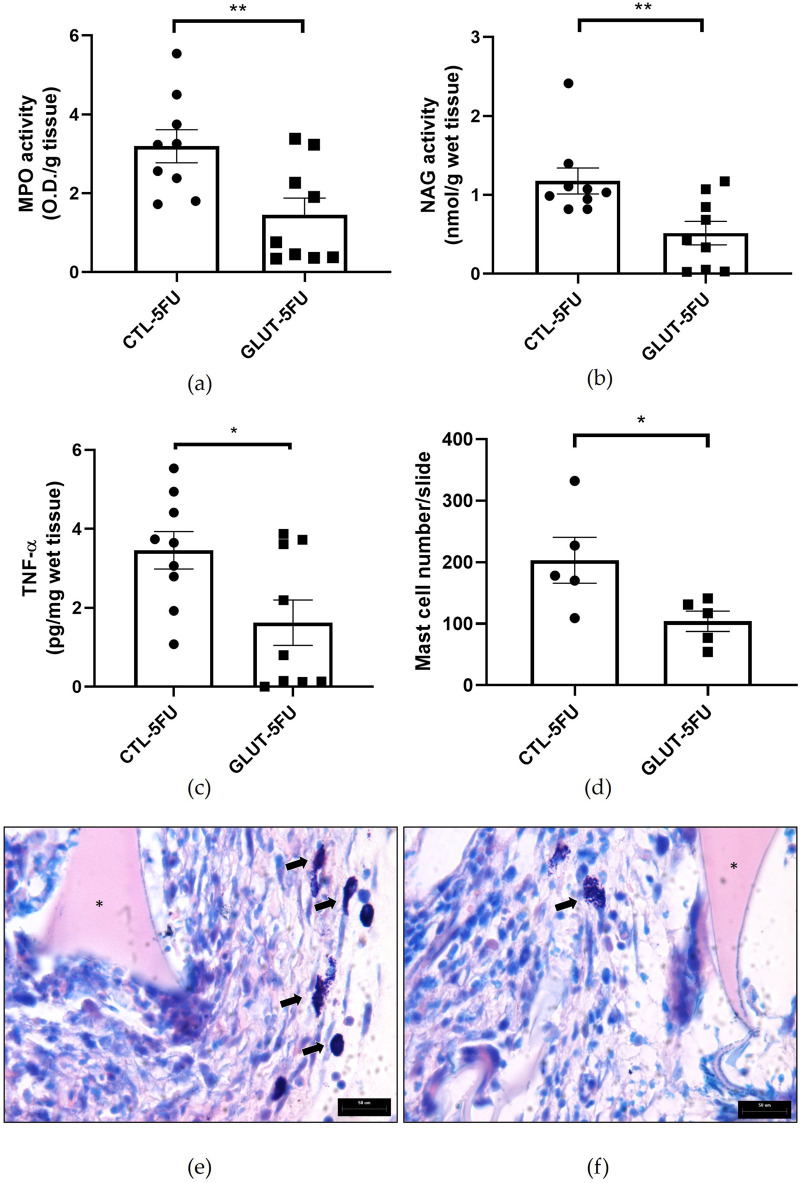
Effects of L-Glutamine on inflammatory markers. The production of all inflammatory markers myeloperoxidase- MPO **(a)**; N‐acetyl‐β‐d‐glucosaminidase-NAG **(b)**; tumor necrosis factor alpha- TNF‐α **(c)** (n = 9); and Mast cells **(d)** counting (n = 5) were lower in the implants after the treatment. Representative photomicrograph of histological sections of the implants control **(e)** versus treated **(f)** stained with Dominici. Values are means ± SEM; *p < 0.05, **p < 0.001, Student’s unpaired t-test. Planapochromatic objective (×100) in light microscopy (final magnification = ×1000). Scale bar, 50 μm.

Further analysis of the inflammatory components of implant-induced fibrovascular tissue were determined by evaluating MPO and NAG activities, and TNF-alpha levels (Figures 6a–c). In addition, the number of mast cells was quantified (Figures 6d–f). L-Glutamine was able to attenuate all the parameters evaluated. The values for MPO were 3.6 ± 0.42 in control versus 1.5 ± 0.42 (O.D/g wet tissue) in the treated group p = 0.010 (n = 9), and NAG values were 1.2 ± 0.16 control versus 0.5 ± 0.15 (nmol/g wet tissue) in L-Glutamine group, p = 0.009 (n = 9). Furthermore, the treatment lowered TNF-α production (pg/mg tissue) in the treatment group (control 3.4 ± 0.47 versus 1.6 ± 0.6; p = 0.025 (n = 9). The number of mast cells/slide was also lower in the treated group, 104 ± 16.5 (n = 5) versus 203 ± 37.0 in the control group, p = 0.041.

### Evaluation of angiogenesis

**FIGURE 7:**
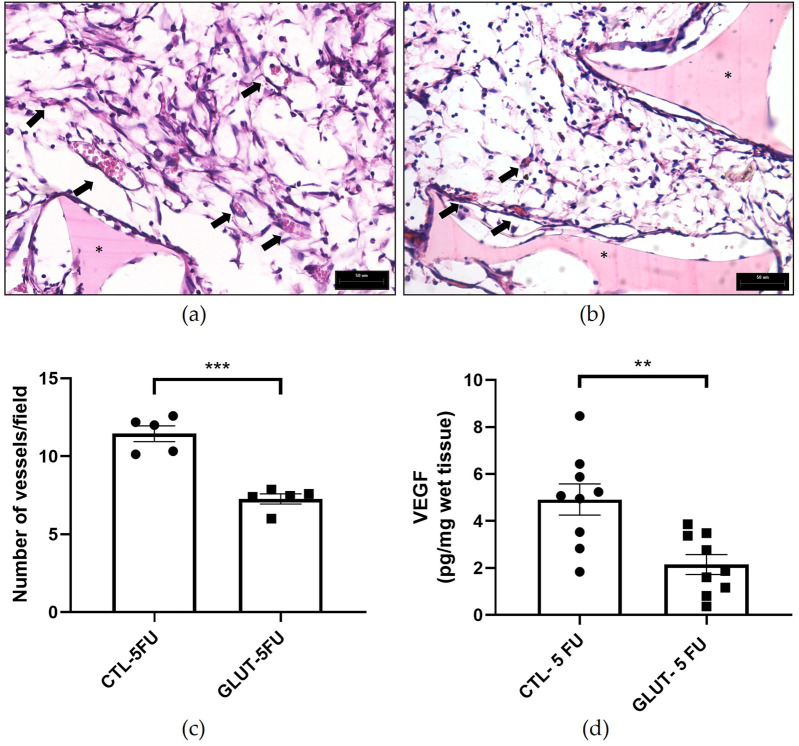
Effects of L-Glutamine on angiogenesis. Photomicrographs of the sponge implant stained with H&E are shown; (**(a)**, control and **(b)**, treated); magnification. Figure 6 **(c,d)** shows the number of blood vessels (n = 5 animals) and VEGF levels (n = 9 animals), respectively. Arrows: intra-implant vessels. The results are expressed as means ± SEM. **p < 0.001, ***p < 0.0001; Student unpaired t test. Planapochromatic objective (×40) in light microscopy (final magnification = ×400), Scale bar, 50 μm.

The supplementation was able to reduce the angiogenic markers evaluated (number of blood vessels, VEGF levels). Histological analysis (H&E) showed that in implants of control animals the number of newly formed vessels was 11 ± 0.5/field (n = 5) versus 7 ± 0.3/field in the treated group (p = 0.0001). VEGF levels were significantly lower in the L-Glutamine-treated group (1.9 ± 0.43 pg/mg, n = 9) compared to the control group (4.9 ± 0.7 pg/mg, n = 9) p = 0.003 (Figures 7a–d).

### Analysis of fibrogenic markers

**FIGURE 8:**
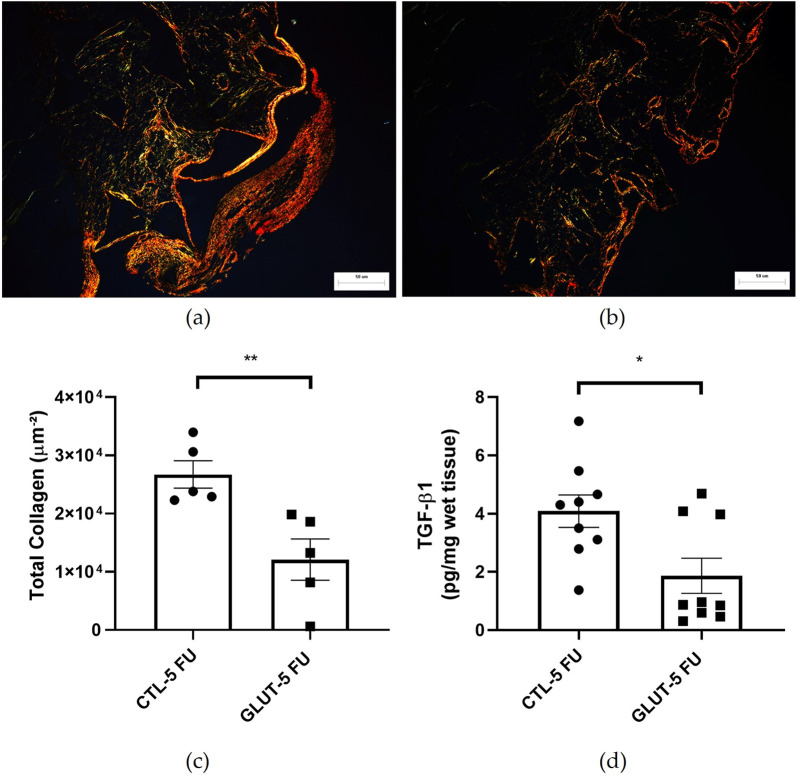
Effects of L-Glutamine on collagen fiber deposition. Representative photomicrographs of intra-implant collagen fibers visualized by Picrosirius red staining showing the control **(a)** and the L-Glutamine-treated **(b)** n = 5 animals. There was a significant reduction in the area of collagen fibers between the groups **(c)**. TGF-β1 levels were lower in the L-Glutamine treated group relative to the control group **(d)** n = 9 animals. Results are expressed as mean ± SEM. *p < 0.05, **p < 0.01, Student’s unpaired t-test. Planapochromatic objective (×20) in light microscopy (final magnification = ×200). Scale bar = 50 µm.

L-Glutamine supplementation was able to attenuate the fibrogenic parameters analyzed (collagen deposition and TGF-β levels). Interestingly, collagen type III (greenish color) was predominant in the treated group. In this group, the fibers had a more fragmented appearance with greater spacing among the fibers compared with the control group (Figures 8a–c). Less total collagen area (Picrosirius red staining) in the treated group was observed 12.10 ± 3.54 versus 26.72 ± 2.35 µm^2^ in the control group p = 0.009 (n = 5) (Figure 8c). The L-Glutamine was effective in reducing the levels of the pro-fibrogenic cytokine TGF-β (control 4.6 ± 0.5 pg/mg) versus 2.8 ± 0.9 in the treated group, p = 0.0156 (n = 9) (Figure 8d).

## Discussion

Peritoneal adhesions, adverse healing processes, are serious medical conditions and major challenges for the health system [1–3]. Although several surgical and pharmacological interventions have been able to attenuate the burden of this condition, none seems to prevent/treat this pathological condition completely. Thus, the search for new therapeutic approaches is warranted [8, 9].

Physiologically, L-Glutamine is directly involved in immunological cell division, acid-base balance, transport of ammonia between tissues, donation of carbon skeletons for gluconeogenesis and participates in processes of recovery from physiological stress and/or catabolic events [10, 14, 37]. It has been reported that this amino acid exerted both anti- and pro- inflammatory, angiogenic and fibrogenic effects on several damaged/injured tissues, thus improving the quality of remodeling in randomized clinical studies [37, 38]. In clinical conditions with high metabolic demand and immunosuppression, such as chemotherapy treatments, burn and critical patients, the body needs a large amount of this amino acid, but its availability is limited. Thus, oral or enteral supplementation of L-Glutamine through diet has been recommended, turning it into a potential therapeutic agent [38–40]. Our objective was to investigate whether this amino acid supplementation would modulate the healing process after peritoneal injury in immunosuppressed mice. The effects of L-Glutamine supplementation were evaluated on mice submitted to chemotherapy (5-FU) and surgical implantation of polyether polyurethane matrix in the abdominal cavity, simultaneously. Both approaches are likely to reflect the clinical scenario of cancer patients that undergo peritoneal surgery with consequent adverse healing. The experimental procedures inflict local and systemic tissue damage requiring further energy supply to comply with the metabolic demand. As reported in previous publications, implantation of the matrix elicited formation of peritoneal fibrosis in which inflammation, angiogenesis and fibrosis were identified [25–28]. The use of 5-FU in our study, based on the work by [31, 33], resulted in a fall of leukocytes (48%), confirming the immunosuppressive effect of the chemotherapeutic agent observed in both humans and experimental animals. It was interesting to observe a progressive body weight gain in the L-Glutamine treated animals whereas body weight loss was seen in the control group, during the time of the experiments. These findings confirm the beneficial effects of L-Glutamine supplementation on systemic metabolism and body weight control [41].

There were also striking differences between the two groups regarding the formation of peritoneal adhesion-like tissue. Visually, the amount of red blood cells was less in the treated group as indicated by the color of the implants (macro analysis). Histological analysis of the implants revealed that the newly formed peritoneal fibrovascular tissue that formed in and around the implants, during the time period examined (8 days), was composed of inflammatory cells, blood vessels, and extracellular matrix proteins. This observation confirms our previous reports that described this model of intra-peritoneal adhesion based on the implantation of polyether-polyurethane sponge matrix [26–29]. However, the number of total cells was less in implants of the animals supplemented with L-Glutamine. These data were corroborated by flow cytometry that demonstrated a marked reduction in the overall frequency of CD45 cells, a 48% reduction in the implants of the treated group. It was interesting that in the remaining leukocyte population in this group, neutrophils, lymphocytes and macrophages represented a higher relative proportion compared with the control group. It has been demonstrated that L-Glutamine exerts anti-apoptotic activity in small intestine injury [42]. This effect may have occurred in our experiments. The number of Caspase-3 positive cells in the control group was higher than in the treated group indicating an anti-apoptotic effect L-Glutamine which may have contributed to the increased number of the inflammatory cell populations in the treated group. The other factor that may have favored the increased number of these cells in the implant of the treated group was availability of amino acid. *In vitro* studies showed that glutamine potentiates the activity of inflammatory cells, promoting activation and function of neutrophils and macrophages and proliferation and differentiation of lymphocytes [43–47]. The other inflammatory parameters analyzed MPO and NAG activities, TNF-alpha levels and mast cell number were decreased in the implants after L-Glutamine supplementation. These soluble and cellular markers are relevant components of the inflammatory response to a number of stimuli, including implantation techniques [26, 27, 29]. Thus, this amino acid favored the anti-inflammatory phenotype observed in our model. The apparent discrepancy between the presence of higher neutrophil/macrophage/lymphocyte infiltration in the peritoneal fibrotic tissue and reduced MPO, NAG, and TNF-α production may suggest immune reprogramming. It has been reported that glutamine shifts polarized inflammatory cells, particularly macrophages and neutrophils, towards their phenotypes and functions [48, 49]. Our results are in line with several reports that demonstrated the anti-inflammatory effects of L-Glutamine supplementation *in vitro* and *in vivo* systems [16, 17, 50, 51].

However, it has been reported that inhibition of L-Glutamine metabolism by an analogue, 6-diazo-5-oxo-L-norleucine, and not its supplementation, accelerated the resolution and repair of acute lung injury induced by intratracheal lipopolysaccharide (LPS) instillation [18].

Furthermore, while L-Glutamine can enhance glutathione production and attenuate oxidative stress, thereby reducing inflammation, it can also serve as a fuel source for rapidly proliferating immune cells, potentially exacerbating inflammatory responses in critical systemic conditions [11, 13, 37]. These seemingly contradictory results are likely to depend on various factors such as, type of injury, phase of the injury, experimental model used. Further investigation will be necessary to establish mechanisms by which L-Glutamine exerted its anti-inflammatory effects on our model.

The anti-inflammatory effects of L-Glutamine supplementation observed in the peritoneal fibrotic tissue occurred in immunodepressed animals. It is well established that early inflammation is essential for tissue clearance and repair initiation and that excessive immune suppression could potentially impair healing. However, the fact that various inflammatory cells (neutrophils, macrophages and lymphocytes) were in higher frequency in the treated group, suggest that L-Glutamine promoted immune reprogramming. Activation and function of these cell populations towards a pro-repair phenotype in immunosuppressed state. Exogenous glutamine has been shown to augment the functions of neutrophils, lymphocytes and macrophages in a rat peritonitis model [52]. Thus, the treatment appeared to have promoted a more balanced immune response, facilitating inflammatory cells reprogramming toward a pro-repair phenotype and supporting physiological tissue remodeling.

Another component of the peritoneal adhesion-like tissue evaluated was angiogenesis. L-Glutamine supplementation was able to decrease the number of blood vessels, and levels of VEGF (key pro-angiogenic molecule). Because in our model, blood vessel formation and inflammation are co-existing processes and L-Glutamine attenuated the latter, down regulation of angiogenesis was expected to occur. Whether this effect was a direct action of L-Glutamine or a consequence of decreased inflammatory process in the implants remains to be determined.

It is clear from the literature that glutamine metabolism is a key factor in angiogenesis. However, its effects on this process are far from clear cut. For instance, pharmacological blockade of glutamine metabolism has been shown to reduce pathological and developmental angiogenesis *in vivo* and endothelial sprouting *in vitro* indicating that endothelial cells depend on extracellular glutamine for proliferation and viability [18, 19]. Our finding showed that rather than stimulating the angiogenic parameters analyzed, L-Glutamine promoted inhibition of VEGF levels and blood vessels number. This discrepant result may be attributed to the type of angiogenesis models used. It is also relevant to consider that the immunosuppression caused by 5-FU may have altered the pattern of glutamine metabolism in our model. However, given that we only studied inflammatory angiogenesis, analysis of angiogenesis in other experimental models is warranted in the future.

Fibroblast activation, proliferation and the products of its activities are essential phases of physiological wound healing, but it is also a process involved in progression of pathological healing and tissue fibrosis. The fibrogenic component of the peritoneal fibrovascular tissue was evaluated through TGF-β production and total collagen (Picrosirious red staining). These markers were reduced by L-Glutamine treatment indicating the anti fibrogenic activity of the amino acid. Picrosirius polarization method is simple, sensitive and specific for collagen staining, particularly useful to reveal the molecular order, organization and/or heterogeneity of collagen fiber orientation in different connective tissues [53]. However, it is not sensitive enough to differentiate collagens subtypes. Thus, further investigation using specific antibodies or molecular markers for collagens types may reveal whether L-Glutamine supplementation modulated the deposition of distinct types of collagen in peritoneal fibrosis. Glutamine has been shown to either up or down regulate fibrosis in several pathological conditions. For instance, the use of L-Glutamine enema reduced inflammation and fibrosis in experimental diversion colitis in Wistar rats [23]. Furthermore, oral administration of glutamine promoted faster skin healing by acting on several stages of healing, such as collagen synthesis, wound contraction and epithelialization, in excision wound model [23]. Several studies, however, have evidenced pro-fibrotic effects of L-Glutamine. One such study revealed that patients with severe fibrosis exhibit elevated serum glutamine levels and increased expression of kidney glutamine synthetase. Deprivation of glutamine metabolism *in vitro* and *in vivo* inhibits fibroblast activation, ameliorating renal fibrosis [21]. Furthermore, inhibition of glutamine metabolism could suppress the activation of hepatic stellate cells attenuating liver fibrosis [25]. It has also been demonstrated that glutamine and its conversion to glutamate by glutaminase are required for TGF-β–induced collagen protein production in lung fibroblasts [54]. A number of factors may explain the divergent and opposing effects of L-Glutamine on fibrosis. One possibility is that in our study, the animals were immunosuppressed, therefore, the cells involved in the fibrogenic response were not under normal physiological state. It is possible that the diversity of tissues, models and experimental protocols used to evaluate the effects of L-Glutamine on fibrosis has contributed to the distinct responses reported.

In our study, using a range of histological and biochemical markers, we demonstrated the efficacy of L-Glutamine supplementation attenuating key components (inflammation, blood vessel formation and collagen deposition) of implant-induced peritoneal fibrosis in 5-FU-treated mice. Glutamine actions in regulating the peritoneal fibrovascular tissue growth is likely to have occurred through activation/inhibition/regulation of a range of molecular signaling pathways. In inflammatory processes, for instance, L-Glutamine has been shown to induce MAPK phosphatase, to inhibit NF-kB and to regulate TGF-B1/SMAD2/3 pathways [17, 50, 55]. In several angiogenic assays, glutamine has been shown to act through mTOR and EGFR/ERK signaling pathways [56]. Potential signaling pathways activated by glutamine such as TGF-β/Smad or mTOR/MTFP1/DRP1 are involved in fibroblast activation in fibrotic diseases [21]. However, we do not provide direct evidence of glutamine actions in our model through these pathways which constitutes a limitation of this study. Certainly, identification of signaling pathways and their specific roles mediating glutamine actions will be necessary for developing glutamine-based therapies in peritoneal fibrosis in immunosuppression conditions.

Another limitation of this study was the fact that we did not include a healthy control group (non-5-FU). This would be relevant to examine the effects of L-Glutamine on fibroproliferative processes in norm-immune animals to rule out the systemic effect of 5-FU on peritoneal fibrosis. However, our main focus was to examine specifically the potential therapeutic effect of L-Glutamine during antineoplastic treatment. In the course of chemotherapy, very often humans and/or animals endures simultaneous tissue damage such as surgeries, tissue replacement, and inflammatory processes. Thus, by inflicting two challenging conditions (immunosuppression and fibrosis) to the animals and examining their systemic and local response to L-Glutamine supplementation, our results clearly show that the supplementation acted systemically by improving body weight (metabolic parameter) and locally by down regulating inflammation, angiogenesis and fibrosis in peritoneal healing tissue in immunosuppressed mice. Thus, our study design was effective in demonstrating, for the first time, the beneficial effects of L-Glutamine on attenuating peritoneal fibrosis in immunosuppressed conditions.

Altogether, our results and published reports highlight the importance of L-Glutamine metabolism on fibrovascular tissue remodeling and the need for further investigation to understand the role of this amino acid in physiological and pathological wound healing.

### Conclusion

In our study, the presence of polyether–polyurethane implants in the peritoneal cavity resulted in the formation of an adhesion-like tissue in mice treated with 5-FU. Oral supplementation of L-Glutamine was able to differentially modulate inflammatory cells of the myeloid lineage towards a predominant anti-inflammatory, anti-angiogenic and anti-fibrogenic phenotype of the peritoneal fibrovascular tissue. These findings suggest that L-Glutamine holds promise for translation into clinical trials as a potential adjuvant therapy in the management of peritoneal fibrosis in immunosuppressed states.

## Data Availability

The original contributions presented in the study are included in the article/Supplementary Material, further inquiries can be directed to the corresponding author.
