# Supplementary figures and images for: L-Glutamine attenuates peritoneal fibrosis developed in 5-Fluorouracil-treated mice
Source: Exp Biol Med (Maywood). 2026 Feb 24;251:10755. doi: 10.3389/ebm.2026.10755 (PMC12971535; doi:10.3389/ebm.2026.10755)

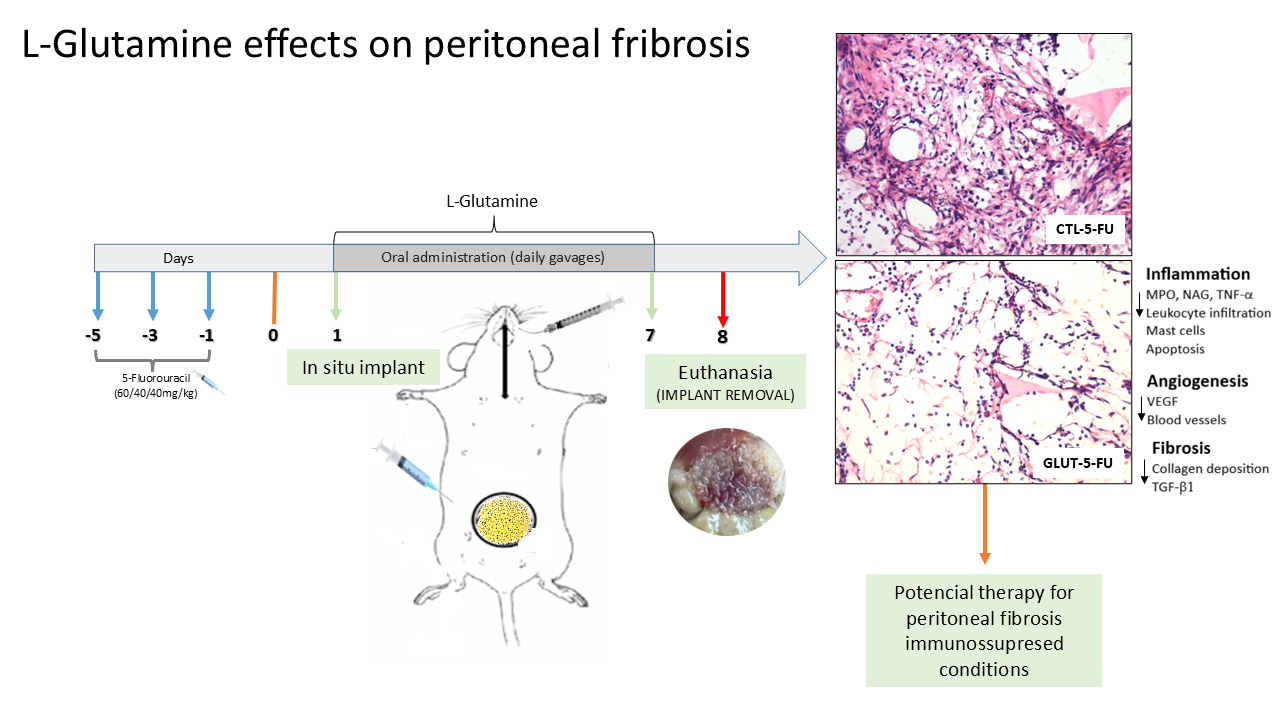

Supplement: Supplementary file 1 [file Image1.tif]
